# Case Report of a Rare but Serious Complication in Anesthesia: Arachnoiditis Following an Epidural Blood Patch

**DOI:** 10.7759/cureus.86295

**Published:** 2025-06-18

**Authors:** David Kalpers, Jérome Jean

**Affiliations:** 1 Anesthesiology, Centre Hospitalier du Luxembourg, Luxembourg, LUX

**Keywords:** anesthesia complication, arachnoiditis, blood patch, epidural analgesia, post dural puncture headache

## Abstract

The epidural blood patch (EBP) is an invasive technique used to plug a leak of cerebrospinal fluid, usually following an iatrogenic lesion of the dura mater. This injection of autologous blood into the epidural space is associated with a number of rare but potentially serious complications. We present a case of arachnoiditis in a young parturient who presented with symptoms compatible with intracranial hypotension due to an accidental dural puncture during an obstetric epidural, and who was treated with two epidural blood patches. The diagnosis of arachnoiditis was suspected based on the neurological symptoms and confirmed by magnetic resonance imaging (MRI) of the lumbosacral region. Monitoring after an EBP and potential treatment options for spinal arachnoiditis are a current topic of discussion in anesthesia.

## Introduction

The injection of autologous blood into the epidural space, commonly known as an epidural blood patch (EBP), is an invasive technique performed by an anesthetist to treat a cerebrospinal fluid (CSF) leak causing post-dural puncture headache (PDPH) symptoms. Often secondary to an iatrogenic lesion of the dura mater, a CSF leak can cause meningeal traction, ultimately inducing intracranial hypotension with a typical symptomatology for patients. The cardinal symptom of this clinical picture is a postural “helmet headache”, which worsens with orthostatism and improves in the supine position. Other symptoms may also be associated with these headaches, such as nausea/vomiting, nuchal rigidity, and auditory and visual disturbances. Conversely, the clinical examination should not reveal pyrexia or signs of neurological deficit, which if present, should lead the clinician to suspect another, potentially more serious, cause for the clinical picture [1].

In the context of a dural puncture, the EBP usually resolves symptoms within 48 hours. Nonetheless, the indication for this technique must be correctly defined, as it has a number of rare but serious potential complications, such as convulsions, sudden blindness, bradycardia or even vagal malaise [2]. The case presented in this article concerns arachnoiditis, which occurred in the days following the treatment of PDPH with two blood patches.

## Case presentation

We present the case of a 30-year-old primiparous female patient with no significant medical history, who presented six hours post an obstetric epidural with typical postural headaches associated with tinnitus, secondary to a suspected accidental dural puncture. In addition, there was no doubt that the catheter tip was intact on removal from the lumbar region. In view of the symptoms, two EBPs were performed 48 hours apart. A first blood patch was performed under ultrasound guidance, and 22 milliliters of autologous blood were injected, with a sensation of lumbar pressure appearing at the end of the procedure. This lumbar pain was isolated (without associated radiculopathy) but persisted until the day after the procedure and was aggravated by sitting. The improvement in PDPH was temporary, and a second blood patch was performed with 30 milliliters of blood injected before the patient described recurrence of the sensation of lumbar pressure. Forty-eight hours after the second blood patch, the PDPH symptoms resolved definitively, and the low-back pain remained moderate and isolated.

Five days after the second blood patch, the patient suddenly complained of increased lumbar pain associated with a stabbing sensation in the lower limbs. Neurological examination revealed pain described as electrical currents, mainly in the S1 and S2 sacral dermatomes of the right leg, exacerbated by right leg flexion. Bilateral plantar cutaneous reflexes were in flexion, and osteotendinous reflexes were normal in both lower limbs. Gait was hesitant with a dodging limp, but there was no evidence of a localized sensory-motor neurological deficit. In addition, the patient remained afebrile.

**Figure 1:**
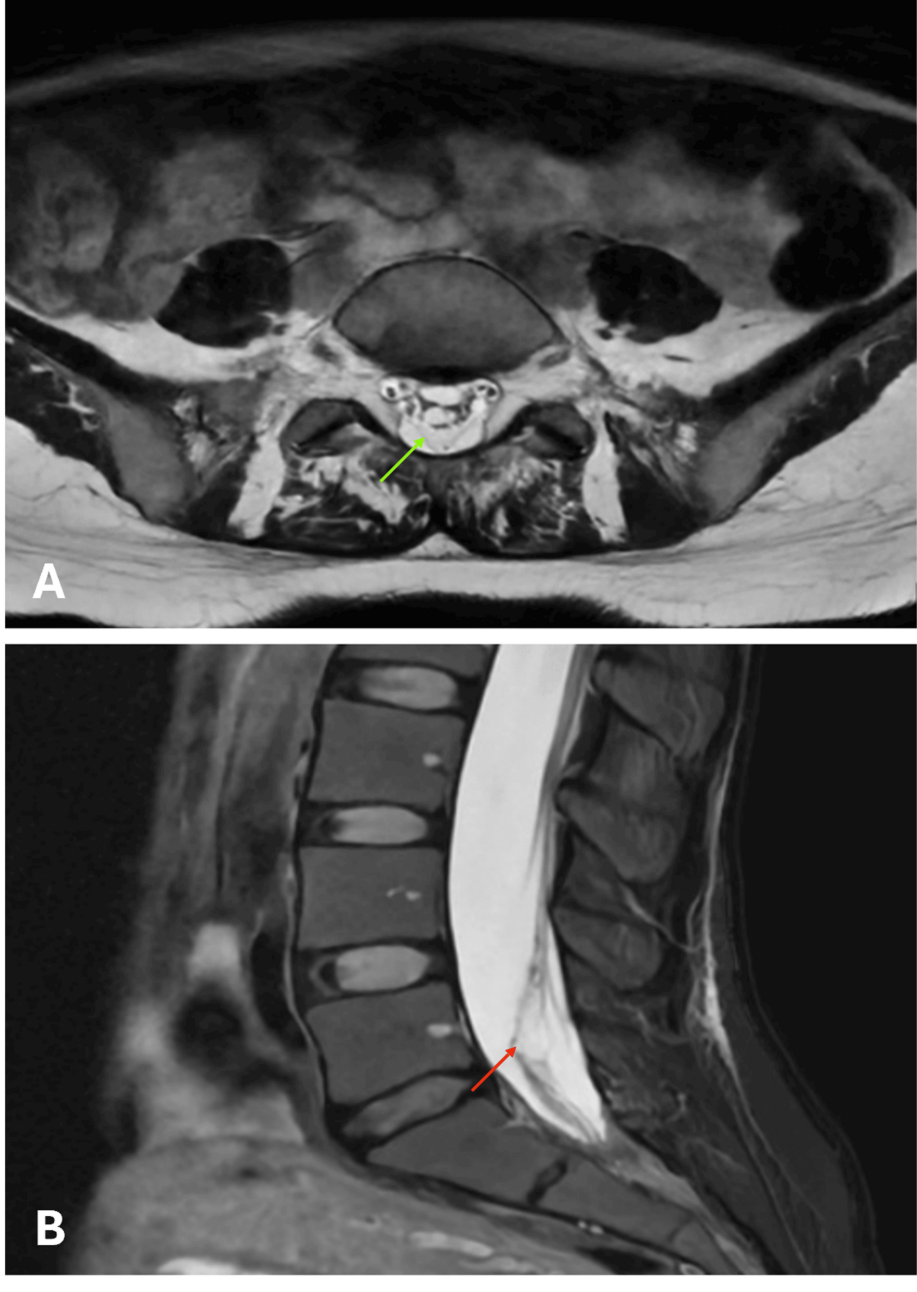
Magnetic resonance imaging, T2 ponderation (A) Transversal section with the green arrow pointing to the fibrosis of the arachnoid. (B) Sagittal section with the red arrow pointing to compression of the nerve roots of cauda equina.

In view of the unusually severe clinical presentation, an MRI of the lumbosacral region was ordered as a matter of urgency. It revealed dorsolateral compression of the cauda equina nerve roots, associated with discrete fibro-cicatricial re-modelling of the L5 and S1 roots bilaterally (Figure 1).

Based on the clinical and radiological findings, a diagnosis of arachnoiditis was made and a pharmacological treatment of analgesics (paracetamol, non-steroidal anti-inflammatory drugs, and gabapentin) combined with corticosteroids (12 milligrams per day of dexamethasone in three divided doses for five days) was initiated. The clinical course was favorable with complete disappearance of symptoms within weeks of the introduction of medical treatment.

## Discussion

Arachnoiditis following EBP is a rare complication, with fewer than a dozen cases reported in recent literature. It consists of an inflammatory response creating adhesions in the collagenous meninges surrounding the nerve roots [3]. The usual causes of this reaction include localized infection (spondylodiscitis), surgery, and trauma, as well as spinal degeneration and neoplasia. It is highly likely that blood in the subarachnoid space, secondary to the EBP, was the cause of irritation of the meninges in the present case [4].

Despite the low rate of serious complications related to EBP, a series of post-intervention symptoms should prompt an MRI to rule out the presence of local complications of the lumbosacral region. These symptoms include persistence of lumbar pain for more than 12 hours after an EBP, the appearance of fever, neurological deficits, muscle weakness, or paresthesia. This will also help exclude other potential treatable emergencies that can be considered in the differential diagnosis, such as epidural hematomas, lumbar disc herniation, or abscesses [3].

There is currently no consensus in the scientific literature on the treatment of spinal arachnoiditis, whatever its etiology. Indeed, while practitioners and authors of articles relating to this pathology agree on analgesic treatment with the usual medications (paracetamol, non-steroidal anti-inflammatories, and tramadol), the use of corticosteroids does not systematically show a positive effect on the evolution of the pathology [5]. Moreover, in the more severe situations, surgery (microlysis and decompressive laminectomy) may become necessary [6].

Like most previously described cases of arachnoiditis, our patient’s symptoms progressed favorably, reporting a full clinical recovery at her last obstetric follow-up [3].

## Conclusions

Arachnoiditis is a rare but serious complication of EBP. Although exceptional, this situation allows us to recall that the EBP presents potential risks and that its indication must be carefully considered before its realization. This incident should serve as a reminder to anesthetists that certain clinical signs and symptoms persisting after EBP should be investigated further by performing emergency imaging. In this regard, MRI remains the reference radiological examination to avoid any erroneous interpretation of a rare diagnosis such as arachnoiditis. Further research is needed to determine whether corticosteroids are a useful addition in the treatment of spinal arachnoiditis.
